# Acceptance and management of stage fright among musicians: a manual of practical strategies

**DOI:** 10.3389/fpsyg.2025.1570280

**Published:** 2025-06-24

**Authors:** Eva Bojner Horwitz, Pauliina Valtasaari

**Affiliations:** ^1^Department of Music, Pedagogy, and Society, Royal College of Music, Stockholm, Sweden; ^2^Department of Neurobiology, Care Sciences, and Society, Karolinska Institute, Solna, Sweden; ^3^Center for Social Sustainability (CSS), Karolinska Institute, Solna, Sweden; ^4^The University of the Arts Helsinki, Sibelius Academy, Helsinki, Finland

**Keywords:** acceptance, manual, musicians, performance, stage fright, stick figures

## Abstract

It has been suggested that stage fright may be musicians’ greatest psychological stressor. This study aims to develop various mental and physical practices that form an easy-to-use “manual” for musicians with stage fright by using *stick figures* and concise, short, instructive texts. The recommended practices were developed based on discussions between expert researchers working with musicians’ stage fright. The manual was tested on a musician to manage unpleasant thoughts, emotions, and physical sensations caused by stage fright. Eighteen steps, rooted in self-awareness and mindfulness, gave the musician a tool to embrace stage fright as a steppingstone to greater self-expression and artistry. The manual with the 18 stick figures will be further evaluated and tested in a controlled bigger sample. The discussions revealed that by observing and accepting one’s feelings without judgment and catastrophizing, it is possible to learn to understand the needs of the body and to respond compassionately. A manual of 18 easy-to-use practical strategies could be one way to deal with stage fright.

## Introduction

Stage fright and music performance anxiety are well-known phenomena that have been studied for several decades. Musicians worldwide and at all professional levels are familiar with the experience. It has even been suggested that stage fright may be musicians’ greatest psychological stressor (Lockwood, 1989; Fernholz et al., 2019).

As early as the 1980s, it was found that classical orchestra musicians experienced greater performance anxiety as compared with musicians working in the genre of popular music. In the Vienna Symphony Orchestra, researchers found that as many as 58% of musicians had experienced nervous stress during the concerts, and 24% had increased levels of tension before the performances (Schulz, 1981). In another study, 25% of musicians reported stage fright, indicating that it was a major problem for 16% of them (Fishbein et al., 1988). Nervous stress and increased bodily tension are commonly reported among classical musicians before entering on stage.

It has been suggested, however, that stage fright should not be regarded as a psychological condition, because it appears to be such a common experience as part of a musician’s life, and a “natural” response to the demands of the job (Brodsky, 1996). It is human nature to be nervous about things that are important and meaningful to us. Physical and mental arousal, as well as stage fright, are familiar reactions for performing artists when they are preparing for a high-quality performance. As we go on stage, the level of stress hormones rises, and the sympathetic nervous system is activated. Nevertheless, stage fright might cause various uncomfortable psychosomatic sensations and anxiety that are stressful for musicians in their everyday work (Kenny, 2011). In the literature, the two terms are often used interchangeably, but they can denote different aspects of anxiety related to performing. “Performance anxiety” is a broad term encompassing anxiety experienced in various performance contexts, including public speaking, music, sports, and other activities where individuals are observed by others. “Stage fright,” on the other hand, specifically refers to anxiety associated with performing on stage, such as in theatrical or musical performances.

For instance, they are sometimes distinguished by saying that stage fright occurs in performing arts like ballet and drama, implying distress in front of large audiences, whereas performance anxiety can occur in various settings, including intimate ones like auditions (Kenny, 2011). Additionally, stage fright often refers to a sudden onset of intense fear, while performance anxiety can build gradually over time.

There are myriad strategies to help musicians cope with unpleasant experiences of nervousness, which we will discuss, but first, we must know why musicians experience stage fright and understand the complex cognitive, emotional, and behavioral structures behind the symptoms. In so doing, we hope to work toward a set of recommendations for a variety of non-pharmacological strategies that can be deployed by performers to cope with stressful situations.

Stage fright is defined as a significant increase in stress hormone levels (adrenaline, noradrenaline and cortisol) and an excitation of the sympathetic nervous system, which the individual perceives as unpleasant (Fredrikson and Gunnarsson, 1992; Theorell et al., 2007). Increased adrenaline levels might cause nervousness, fear, and physical tremors (Vervainioti and Alexopoulos, 2015). During a performance, arousal can have either facilitating (adaptive) or impairing (maladaptive) effects on performance. Adaptive arousal can stimulate the performer’s attention and concentration, while maladaptive arousal can impair the performance (Papageorgi et al., 2007). For performers, there is an optimal level of alertness that improves performance, but when it rises too high it starts to degrade the quality of the performance (Vervainioti and Alexopoulos, 2015).

The onset of performance anxiety is influenced by the individual’s susceptibility to anxiety. This includes intrinsic and extrinsic personal characteristics, cognitive features, comprising factors related to task efficacy (motivation, skills, preparation, and level of commitment) and factors related to the performance environment (audience and venue characteristics) (Papageorgi et al., 2007). Moreover, musicians’ self-imposed pressures may have an influence, such as insufficient preparation for performances and excessive nervous excitement (Kenny et al., 2014).

Stage fright is also a significant social phenomenon, which is why it is important to approach it more broadly than as an individual issue. It can be caused by fear of public criticism (Jakucowicz, 2016), and musicians may be more nervous about evaluation from their well-known colleagues than an unknown audience (Valtasaari, 2023). In contrast, a positive communication culture, and mutual respect are protective factors which offset music performance anxiety (Spahn, 2015). Social support has also been suggested to alleviate work-related stress in musicians and to precipitate lower levels of anti-stress hormones (Theorell et al., 2007).

Stage fright can also be managed by changing one’s attitudes (Juncos and Markman, 2016), while mental training can also help with better concentration, self-perception, experiences of self-efficacy, and more flexible coping when failures occur (Hatfield, 2016). Relaxation exercises (Vaag et al., 2016), breathing exercises (Studer et al., 2011), exposure training, suggestive mental training, different cognitive strategies (Spahn et al., 2016), and psychoeducational mindfulness-based interventions have been found to alleviate the negative experiences of performance anxiety (Steyn et al., 2016). Alongside psycho-physical exercises, it is particularly important to develop practice strategies by planning reasonable schedules, recording one’s own playing, setting realistic goals, and receiving constructive feedback and advice to improve one’s own playing (Steyn et al., 2016).

In this article, we introduce a range of mental and physical practices to help musicians manage the natural but often unpleasant thoughts, emotions, and physical sensations caused by stage fright. The recommended practices were developed based on the discussions between experts working with musicians’ performance anxiety and stage fright.

In the research literature, there is scarce information on how to cope with stage fright, and often different competing theories are presented, such as embodied theory and mirror neuron theory (Bojner Horwitz et al., 2023). This article presents a set of practice recommendations, based on embodied knowledge and experiences that resulted from two research experts working with musicians experiencing stage fright.

The study aims to present a range of mental and physical practices that form an easy-to-use “manual” for musicians with stage fright. Our goal is to present an easy-to-reference guide, using *stick figures* and concise short instructive texts.

## Methods

Data was collected through open-ended discussions between two researchers working with musicians and music students experiencing stage fright. The researchers were experts in the cognitive and emotional experiences of artists, having spent many years working with musicians. The discussions were held on six separate occasions and were facilitated via Zoom (three times) and in-person (three times). Each session lasted 90 min, and all took place during 2024.

The manual was created out of many years of experience working with both music students and musicians with stage fright. The researchers have come to embody the experiences of both students and musicians, having become experts through gathering data that is both broad and deep. The individual experiences of the researchers, respectively, have been very rich and offered detailed understandings and yielded strong affection for the craft of the artists.

The discussion was loosely structured around the *following questions:*

*1 What kind of experiences do we have from our work with stage fright?*
*2 What kind of symptoms are often part of stage fright?*
*3 What do we do to be able to manage the consequences of stage fright?*
*4 What kind of steps need to be taken care of when entering a stage with stage fright?*
*5 What kind of experiences do we have when taking care of stage fright?*
*6 How do we know that the musician is well prepared for being on stage*?

Based on the notes from the discussions, the two experts created a list of 18 themes. Each theme was presented and commented on from the researchers’ pre-understanding of working with stage fright. After integrating the comments, a draft of the 18 themes from the researchers was put together as a whole.

Following the data generation, the researchers illustrated the themes into figurative drawings trying to be as open-minded and creative as possible. After creating drafts of different figurative steps and testing them with a musician, three different intentions/strategies (Feel, Act, and Result) were added to each theme (Table 1). The stick figures were re-drawn by a professional artist, and the results from the researchers and the musician were added to the manual as themes and as five categories (see colors in Table 2). The following categories: (1) awareness and attention, (2) stress and relaxation, (3) self-reflection, (4) visualization and goal setting and (5) recalling positive experiences were developed and will be further developed and discussed.

**Table 1:** The table presents the 18 themes related to each one of the stick figures.

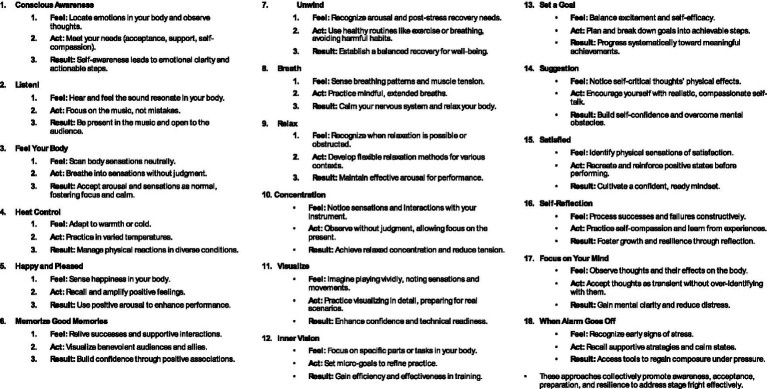

Each theme is divided into the intentions Feel, Act, and Result. (The colors represent the different categories, see Table 2).

**Table 2:** The table presents the five categories related to taking care of stage fright.

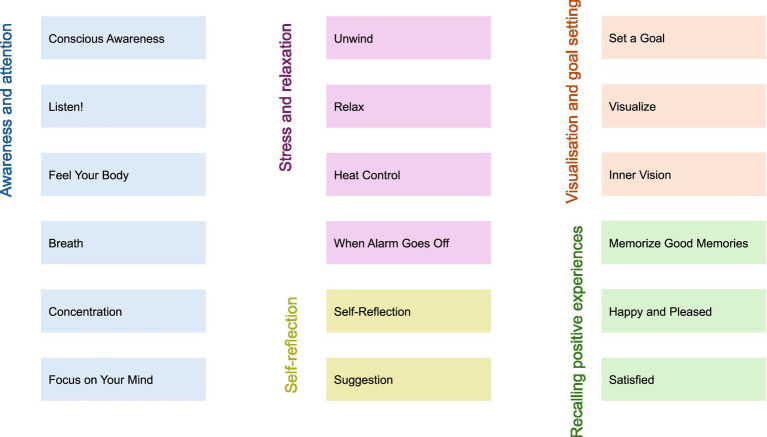

The five categories are: Awareness and attention (blue), Stress and relaxation (pink), Self-reflexion (yellow), Visualisation and goal setting (pink), Recalling positive experiences (green).

## Results

**Figure 1:**
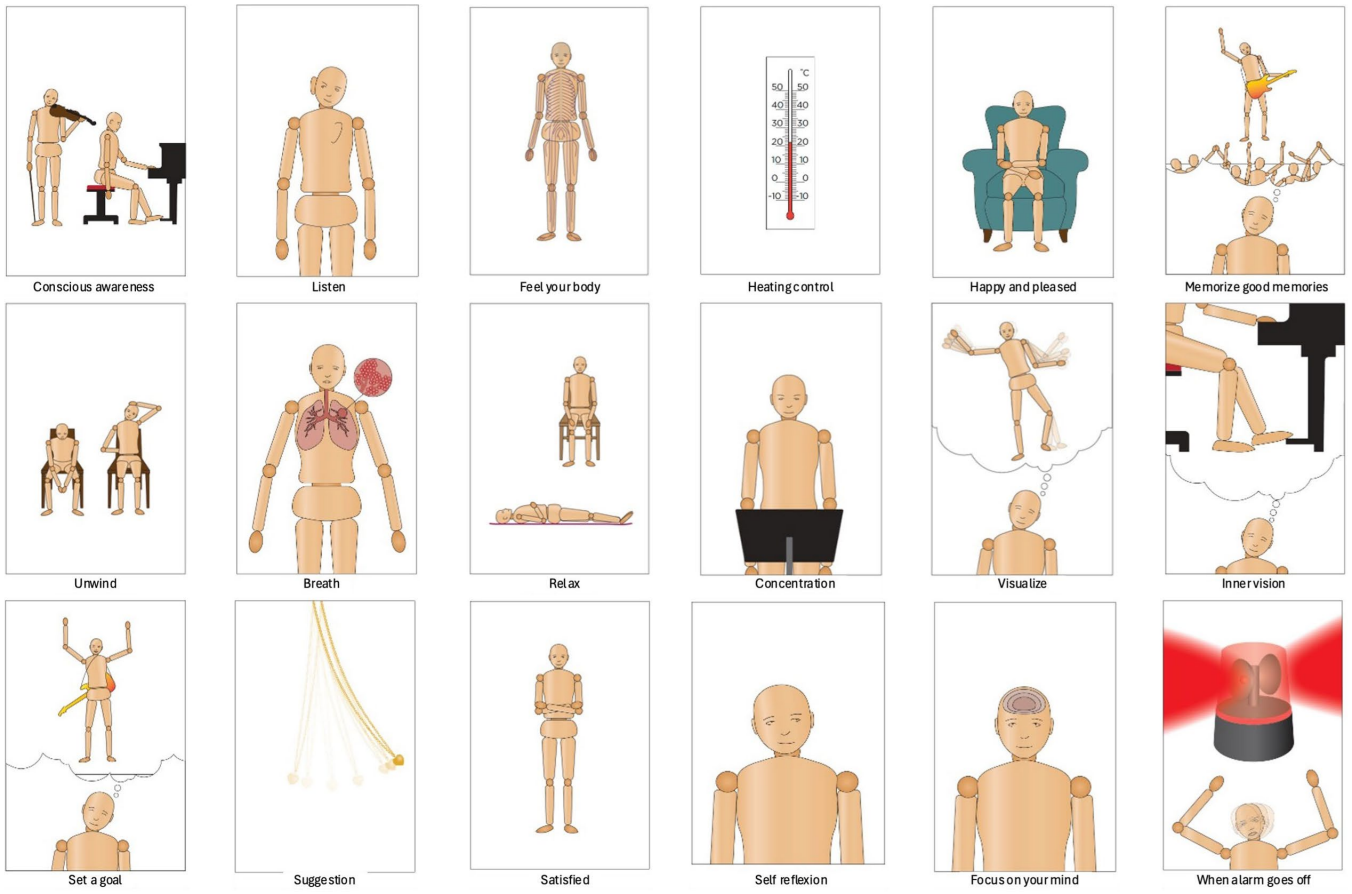
The figure shows 18 stick figures professionally made from researchers’ drawing drafts. The different figures represent mental and physical practices in how to cope with stage fright.

In the following section, we introduce the 18 themes that were part of the researchers’ mapping of ideas helping musicians cope with stage fright (see Figure 1). Table 1 represents the different themes that were added to each stick figure in the manual (see below). According to the researcher’s reflections on their experiences working with musicians, addressing stage fright begins with conscious awareness of the sensations and emotions in your body. The discussions revealed that by observing and accepting one’s feelings without judgment and catastrophizing, it is possible to learn to understand the needs of the body and to respond compassionately. Listening attentively to your instrument and the music you create helps anchor you in the present moment, fostering deep concentration and connection. Feeling your body and accepting its signals as normal can transform anxiety into a tool for focus and energy. Practical techniques such as controlling the ambient temperature, recalling positive memories of previous performances, and visualizing success create a foundation for resilience.

The journey toward calm continues with cultivating relaxation and mastering breathing techniques. This calms the nervous system and prepares the mind for peak performance. Concentration on pre signals from the body and visualization deepen your mental and physical connection to your music, enabling you to perform with confidence and flow. Setting realistic goals and practicing positive self-suggestion enhance your self-efficacy. Reflecting constructively on mistakes allows for continual growth. Developing self-compassion and adopting a balanced mindset help performers approach challenges with grace. Focusing on your thoughts and learning to observe them without attachment can bring clarity and calm, empowering you to face even the most daunting moments on stage. Finally, recognizing and addressing the body’s alarm signals before they escalate ensures that you maintain control and composure. These 18 steps, rooted in self-awareness and mindfulness, gave the musician the tools to embrace stage fright as a steppingstone to greater self-expression and artistry.

## Discussion

Stage fright is a common and deeply human experience that affects performers across disciplines. While it can be daunting, it also presents an opportunity for growth and self-discovery. Understanding and addressing stage fright involves more than just overcoming nervousness; it is about building a meaningful relationship with your body, mind, and emotions. We argue that by exploring the physical, emotional, and cognitive aspects of stage fright, performers can transform these moments of vulnerability into powerful experiences of self-expression and connection. The steps presented in the manual provide a holistic guide to understanding and managing stage fright and going beyond this to help performers cultivate confidence, resilience, and joy in their craft.

In addition to individual experiences and practices, it is equally important to focus on social wellbeing and psychological safety in orchestras and other musical communities. Only by doing this, can we create conditions in which musicians can thrive. Experience of compassion and social support in general have a significant impact on our wellbeing and health (Bojner Horwitz et al., 2023; Tahirbegi, 2022). It is likely to relieve physical pain and stress, helps us to learn new things faster, increases positive emotions, and serve as an important source of inner motivation. Psychological safety raises our creative potential and even offers an opening for self-transcendence (Guyon et al., 2020).

One important finding is the inclusion of *long-term exercises* of the subjects. Work often begins with acceptance of the phenomenon as part the entire life span of the musician and therefore it is important to routinise self-improvement strategies. To be able to use the skills that we have outlined during an episode of stage fright, it would be important to practice them regularly. The more you have trained those skills, the faster and easier you can use them in real situations. This may also function as a preventive action. We would encourage the students to practice the skills regularly so that they have a flexible” toolbox” at their command. Having undertaken regular practice, a manual booklet can act as a quick reminder of useful actions when they need it, before a performance on their own or together with someone else, before a performance, or during practice. We know that the role of mentoring relationships can be highly valuable in the learning process (Valtasaari, 2023) and therefore could also be an important part of the manual. The exercises might be even more effective if one has a mentor/teacher/colleague with whom to practice and reflect on experiences before and after performances.

According to Vygotsky (1978), people learn even the most demanding skills more quickly when they learn in a safe, supportive environment. The type of teacher, mentor or coach with whom a young musician can learn to perform safely is important. The further a musician develops professionally, the teacher-student relationship is often replaced by peer mentoring relationships. Arousal of the body and mind before a performance should be seen as a normal stage of a musician’s work, and the more it is openly discussed also in musicians’ communities, the less there is a false stigma of weakness or unprofessionalism around it.

Further to impact on performance, for a musician, stage fright may also limit the effectiveness of social networking, which has negative consequences on an individual’s opportunities and goals, according to Maslow’s “theory of needs” (Korp, 2016). Moreover, there is a broader health benefit to alleviating anxiety. In spreading knowledge about self-help methods in health and social care this offers preventive measures for at-risk groups (Korp, 2016). Early interventions in educational contexts and workplaces in managing a range of forms of performance anxiety and can therefore have a positive impact on health and wellbeing, seen from a larger societal perspective.

Bandura (1997) argues that an individual’s past experiences of success reinforce his or her sense of capability and how he or she will deal with future challenges. Also, the experience of hope is related to the individual’s perception of the causality of the outcome of their individual actions and the experience of being as an active agent to achieve the desired outcome (Cheavens and Guter, 2017). What is significant in the experience of hope is an individual’s ability to clearly articulate their own goals and develop alternative pathways to achieve them, but also to act in a goal-directed way and maintain their own motivation to use the planned strategies (Snyder et al., 1991; Snyder et al., 2002; Snyder et al., 2003; Marques and Lopez, 2017). When the goals are clear but flexible, alternative pathways and intrinsic motivation help the individual to persevere even when difficulties or obstacles arise (Marques and Lopez, 2017). Our curated exercises give practical steps for trying out different alternative paths. Regular practice can strengthen musicians’ experiences of hope and help them achieve positive cycles of success from one performance to the next and to make performing more confident and rewarding.

Besides supporting musicians’ experiences of hope and self-efficacy, this manual offers insights into compassionate self-awareness. Resilience to face one’s mistakes and failures is also important for the wellbeing of the human mind (Dahl, 2025). Over the musician’s life span, they will have very different performances depending on their life situation and their current psycho-social resources. As everyone experiences underperformance at some point, it is important to develop self-compassion skills so that one’s self-esteem does not depend on individual performance. According to Neff (2011), self-compassion skills increase acceptance, the ability to put problems into perspective, and the ability to take responsibility for one’s actions.

## Limitations

The focus of this research was to bring together insights from professional experience, harnessing the knowledge of the researchers who have worked across multiple projects related to the present study. Furthermore, the authors played multiple roles in the research – teachers, supervisors and researchers which offered different perspectives. This was therefore a practice-driven study, which yields direct implications for those affected by the experience of stage fright. In other work that we have conducted around artistic practices (Korosec et al., 2024; Korošec, 2024) we focus on developing theory much more strongly. This focus also dictated the more interpretive method that was followed, which differs from the more common designs of studies that focus on theory development. These two forms of research are complementary and together give us a better picture of the benefits that can be offered to practitioners.

In the future, it would be valuable to follow up on the insights generated in this study and to evaluate their impact in practice. Such studies might also test the manual in different fields of artistic endeavor, or artists working under different conditions (for example, freelance, in orchestras, students). During such evaluation, there is scope to further the findings presented here through developing a more defined theoretical structure and categorization around self-efficacy and self-awareness.

## Data Availability

The raw data supporting the conclusions of this article will be made available by the authors, without undue reservation.
